# Social Modeling of Food Intake: No Evidence for Moderation by Identification With the Norm Referent Group

**DOI:** 10.3389/fpsyg.2019.00159

**Published:** 2019-02-01

**Authors:** Jinyu Liu, Suzanne Higgs

**Affiliations:** School of Psychology, University of Birmingham, Birmingham, United Kingdom

**Keywords:** modeling, social identity, food intake, social influence, eating

## Abstract

Normative information has a powerful effect on food intake and food selection. People tend to use the eating behavior of others as a reference for their own eating behaviors and match their intake to an eating partner. This is known as social modeling. There is some evidence to suggest that people are more likely to model a norm when it comes from an in-group than when it comes from an out-group, but whether the strength of identification with a norm referent group moderates modeling of intake has yet to be examined. The current paper presents the results of two studies that investigated whether modeling of intake is moderated by strength of identification with the norm referent group. In Study 1, we recruited 90 female students from the University of Birmingham (UoB) (mean age = 21). Students were allocated to either a low norm condition (presented with a sheet that presented a low cookie intake of previous participants) or a high norm condition (presented with a sheet that presented a high cookie intake of previous participants), or a no norm condition (control group without the sheet containing information about previous participants’ cookie intake). Students also completed a questionnaire on their identification as a Birmingham student and cookie intake was assessed. In Study 2, we recruited 84 students (mean age = 21) who were randomly allocated to one of two conditions (a group presented with a high norm for vegetable intake or no information about a vegetable intake norm). Strong modeling effects were found across both studies but the extent to which the participants identified as a Birmingham University Student did not moderate these effects. The moderating effect of social identity on modeling of eating might be context-dependent.

## Introduction

Much evidence has accumulated to suggest that social context is a powerful influence on eating behavior (e.g., Herman et al., 2003; Robinson and Higgs, 2013; Robinson et al., 2014; Cruwys et al., 2015; Higgs, 2015; Higgs and Thomas, 2016). Individuals determine what is appropriate in terms of eating behaviors by looking to social and environmental cues (Nisbett and Storms, 1974). Cues such as the intake of others or portion sizes indicate what is normative consumption and people adjust their eating to align with the norm, which is known as modeling (Roth et al., 2001; Herman et al., 2003). Modeling behavior has been studied widely in the laboratory and in a typical social modeling study, participants’ eating behaviors are observed in the presence of someone else (a confederate of the experimenter who acts as the eating companion and eats as directed by the experimenter) (Herman et al., 2003). What has been found is that the intake of the participants is influenced by that of the confederate (e.g., de Luca and Spigelman, 1979; Conger et al., 1980; Goldman et al., 1991; Hermans et al., 2009, 2010; Feeney et al., 2011; Cruwys et al., 2015). In general, people eat more when their eating companions eat a lot, while they eat less when their eating companions a small amount (Herman et al., 2003; McFerran et al., 2010).

Modeling of food intake has also been reported in situations where there is no person present and the eating norm is communicated by alternative means. In the remote-confederate paradigm, information is provided about the behavior of other participants in the experiment (Roth et al., 2001; Pliner and Mann, 2004; Leone et al., 2007; Feeney et al., 2011; Bevelander et al., 2013; Florack et al., 2013; Robinson et al., 2013; Vartanian et al., 2013). The effect of a remote confederate model on eating behavior has been reported to be similar to that of a live confederate model (Feeney et al., 2011). For example, participants were found to consume significantly more cookies if they were exposed to a high intake norm (information that previous experiment participants ate a large amount of cookies) compared with participants who were exposed to no normative information (Robinson et al., 2013).

There is evidence that normative effects on eating can be enhanced when people identify with the norm referent group (Louis et al., 2007; Stok et al., 2012). For example, participants who saw a majority descriptive norm conveying that most group members consume sufficient vegetables, subsequently self-reported eating substantially more vegetables than those who saw a minority descriptive norm conveying that only a few group members eat sufficient vegetables, but only when they strongly identified with the norm referent group (Stok et al., 2014b). However, Banas et al. (2016) reported that participants who strongly identified with a norm referent group behaved in a manner that was opposite to the depicted norm. These results suggest that under some circumstances people who identify highly with a social group may engage in behavior contrary to that of other group members (Banas et al., 2016). Using a live modeling study design, Cruwys and colleagues found that participants were more likely to model food intake if the normative information was provided by a member of social in-group (a student from the same university), but they were less likely to model if the information is from an out-group (a student from a different university) (Cruwys et al., 2012). Here we extend the literature on modeling by aiming to establish whether strength of identification with the norm referent group moderates modeling in a remote confederate design. Given the reported similarities between the live model and remote model designs (Feeney et al., 2011) we would expect similar moderating influences, but this has yet to be established.

Evidence suggests that people readily model the consumption of palatable, energy dense foods, such as cookies (Roth et al., 2001; Robinson et al., 2013; Vartanian et al., 2013), chocolate M&Ms (Robinson et al., 2011) and popcorn (Cruwys et al., 2012). However, there is little evidence concerning the modeling of nutrient rich foods, such as vegetables and some evidence to suggest that people may not model of intake of ‘healthy’ or unpalatable foods (Goldman et al., 1991; Pliner and Mann, 2004). To date, only one live modeling study has involved consumption of vegetables (Hermans et al., 2009). Participants consumed more vegetables if they were exposed to a peer eating a large amount of vegetables than if they were exposed to a peer eating a small amount or no vegetables. Although a modeling effect on vegetable intake was observed, the effect size was small and it is unclear whether there are any moderators of the effect.

The aim of the present studies was to find out whether strength identification with the norm moderates modeling of food intake. Two studies were conducted examining (1) the modeling of a palatable, energy-dense snack (cookies) and (2) modeling of a low-energy-dense nutrient rich snack (vegetables). The first aim was to investigate modeling of intake of both high calorie and nutrient rich foods (Roth et al., 2001; Hermans et al., 2009; Robinson et al., 2011, 2013; Cruwys et al., 2012; Vartanian et al., 2013). A second aim was to examine evidence for the moderating influence of social identity on intake modeling. For both studies, it was hypothesized that (1) participants would eat more snack foods when they were exposed to high normative information than when they were exposed no normative information and that (2) they would consume fewer snacks when they were exposed to low intake normative information than when they were exposed to no normative information (Study 1 only). It was further hypothesized that any modeling effects would be stronger when participants strongly identified as a member of the norm referent group.

## Study 1

### Methods

#### Participants

Ninety students at University of Birmingham (all females) with a mean age of 21.2 years (*SD* = 2.5) were recruited through advertisement via online portals and posters around campus. Based on calculations using GPower 3.1.0, at 85% power with a *p* < 0.05 and a medium effect size (f^2^) of 0.15 in a linear multiple regression test, a minimum sample of 76 participants was needed for a moderated multiple regression analysis (with 2 tested predictors). We recruited 94 University of Birmingham students to the study. After applying the exclusion criteria, 90 students remained in the sample (mean age of 20.5 years, *SD* = 3.2). One participant was excluded because she was underweight and three participants excluded because they guessed study aims. There was no data collection after data analysis. A *post hoc* test revealed that the study was powered to detect a medium effect size assuming an alpha significance criterion of 0.05, and 91% power criterion.

Students voluntarily signed up for participation. All students were compensated with either course credits or a $5 cash upon the completion of the study. Only females were recruited because of evidence that modeling effects may be stronger for women than for men (Vartanian et al., 2007). Based on the remote and live modeling studies conducted by Robinson et al. (2013) and Robinson and Higgs (2013), a cover story was used to disguise the aims of the study. The adverts suggested that the study was about “Cookie Taste and Mood Status.” Smokers and those with food allergies were excluded from participation. The study was conducted according to the guidelines laid down in the Declaration of Helsinki and was approved by the University of Birmingham Research Ethics Committee.

#### Design

The study used a between-subjects design, with two conditions: message type (high intake norm vs. low intake norm vs. no intake norm) and student identity as a continuous variable. Participants were randomly allocated to one of the three message conditions.

#### Remote Confederate Manipulation

In the experimental conditions (high intake norm and low intake norm), participants were exposed to a sheet containing fictitious information about previous participants. At the top of the sheet, participants saw the following information: ‘Participation sheet- UoB students (University of Birmingham). Please fill in your information below if you are willing to take part in the study.’ The sheet displayed information about five previous participants including their name, age and the amount of cookies eaten. The level of cookie intake was based on previous research and the results of a pilot study. Firstly, Robinson et al. (2013) reported that female psychology students consumed about 4 cookies on average in their experiment. The high norm in that study was approximately 10 cookies. Secondly, Roth et al. (2001) and Vartanian et al. (2013) displayed 13 to 15 cookies in the high-intake condition in a remote modeling study. More importantly, the data we collected from our pilot study suggested that female students ate 5 cookies on average. Based on the above data, and to ensure a large difference between the high norm and low norm condition, in the high norm condition, the amount of cookies listed on the sheet was around 13–15 (15,13,13,14,15) cookies for the high norm condition and 1–2 (2,2,1,2,1) cookies in the low norm condition.

#### Food

The cookies were “Sainsbury Maryland Chocolate Cookies.” All cookies were served in bowl and a glass of water and napkins were also provided. Each bowl contained 20 cookies with a total pre-selection weight of around 210 g.

#### Measurements

In this study, we report all measures and manipulations. Participants’ baseline hunger (and fullness and desire to eat) were measured on a 100 cm visual analog (VAS) scale. Participants were asked to indicate “how hungry are you right now” between “not at all” and “extremely.” To corroborate the cover story, participants were given visual analog scales (100 cm) to rate their mood status (including how happy, alert, drowsy, light-headed, anxious, nauseous, sad, withdrawn, faint, thirsty are you right now). To assess the strength of student identity, we modified the multicomponent identification questionnaire with 14 items (a 7-Likert scale) which was derived from Leach et al. (2008) (Cronbach’s alpha for the current study = 0.85). Participants stated their identity between ‘strongly disagree’ and ‘strongly agree’ (e.g., I am glad to be a student at University of Birmingham). We also added an additional subcategory of identity of ‘motivation’: that is to what extent students are motivated to be identified as a student at University of Birmingham (e.g., I want to see myself as a UoB student/identify with other UoB student). The data related to motivation category are not reported in this paper. Usual snack food intake was measured by two items (how many high energy dense snack foods do you normally eat a day/think back carefully, how many high energy dense snack foods did you eat yesterday). This was based on previous research suggesting that habitual intake may moderate norm following on food selection (Robinson et al., 2014). Next to the questions about high energy dense snack foods, the participants were given information about what kinds of foods are in this category of high energy dense foods (e.g., biscuits, chocolate bars). The liking of cookies was also rated on a 100 cm scale from 0 (not at all) to 100 (very much). To assess dietary patterns (hunger, disinhibition and cognitive restraint of eating), participants completed the Three Factor Eating Questionnaire (TFEQ) (Stunkard and Messick, 1985). There were also demand check questions to find out if participants were aware of study aims and whether participants noticed the norms on the information sheet.

#### Procedure

All experimental sessions took place between 10:00–12:00 and 14:00–18:00 on weekdays. The participant was informed to refrain from eating for 2 h prior to the experimental session. On arrival, the participant was informed about the study details and asked to provide written informed consent. Then she was given the participant sheet and she filled in her own information, such as age and gender. In the high norm and low norm conditions, the participant saw an information sheet showing either high or low cookie intake of previous participants. In the no norm condition, no information was provided about the cookie intake of previous participants (the cells were left blank). After that, the experimenter removed the information sheet and served the cookies. The participant was told to eat as much as she liked in 10 min while completing the taste ratings. The participant also completed the appetite and mood scales before and after eating. Immediately after the taste test, the participant was asked to complete the habitual food consumption questionnaire, the student identification scales and the TFEQ. The participant was also asked to guess the aims of the study, report whether she was aware of the information on the participant sheet, to write down the number of cookies eaten if she remembered it and report whether that information affected her intake in the study or not. Before leaving, the participant’s weight (kg) and height (cm) were recorded. Finally, the participant was debriefed and thanked for her time. Participants’ cookie intake was measured in both grams and number of cookies by the experimenter.

### Analysis Strategy

Before performing the main analysis, we first examined whether the conditions differed in terms of participant age, baseline hunger, BMI and cognitive restraint. In addition, we also examined whether the conditions differed in terms of reported habitual snack food intake and liking of cookies and whether those two variables were correlated with cookie intake. The two items measuring habitual snack food intake (snack food per day and yesterday) were positively and significantly correlated (*r* = 0.64, *p* < 0.001). Therefore, we calculated the average scores for those two items as the habitual snack food intake. A moderated regression analysis was conducted using the PROCESS macro for SPSS to compare the effect of message type and student identification (average total identity scores), and their interaction on cookie intake. Any significant interactions were further examined with follow-up *post hoc* tests.

### Results

#### Manipulation Checks

All participants in the norm conditions (*N* = 60) reported that they remembered the norm information given and correctly reported the number of cookies eaten by previous participants as either 13, 14, or 15 cookies for the high norm condition and 1 or 2 cookies for the low norm condition. Participants in the control condition (*N* = 30) reported no awareness of normative information.

#### Participant Characteristics and Baseline Measures

There was no difference in age, baseline appetite ratings, BMI, TFEQ scores and liking of cookies and habitual snack food intake across three conditions (Table 1). For the whole sample, the appetite ratings were consistent with the participants being moderately hungry: baseline hunger score *M* = 54.1, *SD* = 27.3, baseline fullness score *M* = 27.9, *SD* = 21.0 and desire to eat scores *M* = 63.3, *SD* = 24.0. The mean restraint eating score was 8.9 (*SD* = 5.1) which suggests that dieting tendencies were not high in the population. The average liking of the cookies was 71.6 (*SD* = 21.9) across all conditions, which suggests the cookies were liked.

**Table 1:** Participant characteristics in three conditions.

	No norm (*N* = 30)	Low norm (*N* = 30)	High norm (*N* = 30)
Age (years)	21.6 (3.3)	20.8 (2.1)	21.2 (2.0)
BMI	21.8 (2.0)	21.1 (1.6)	21.3 (2.1)
Ethnicity	White = 14 Asian = 10	White = 15 Asian = 10	White = 17 Asian = 8
Baseline hunger (0–100)	57.1 (25.0)	55.0 (28.8)	50.3 (28.4)
Baseline desire to eat (0–100)	67.3 (19.4)	63.6 (28.2)	59.1 (23.9)
Liking of cookies (0–100)	75.1 (17.7)	75.1 (21.8)	64.7 (24.7)
Restrained eating (0–20)	9.4 (5.6)	8.0 (5.2)	9.3 (4.5)
Habitual snack intake (serving/per day)	1.1 (1.0)	1.2 (0.8)	1.4 (1.0)


*Results shown as mean and (SD).*

#### Correlations Between Baseline Characteristics and Cookie Intake

There were no significant correlations between baseline hunger, baseline fullness, baseline desire to eat, liking of cookies, age or BMI and cookie intake (all *p* > 0.05) and so there was no need to control for these variables. Habitual snack food intake was significantly correlated with total cookie intake (*r* = 0.22, *p* = 0.04) and so we controlled for habitual snack food intake in the subsequent analyses.

#### Cookie Intake

We conducted linear regression using PROCESS macro for SPSS version 3.2. Using indicator coding for the multicategorical independent variable, we examined whether the amount of cookies eaten was predicted by norm condition (Hayes and Montoya, 2017). We also included centered identity scores and centered identity score ^∗^ condition interactions in the regression model.

**FIGURE 1:**
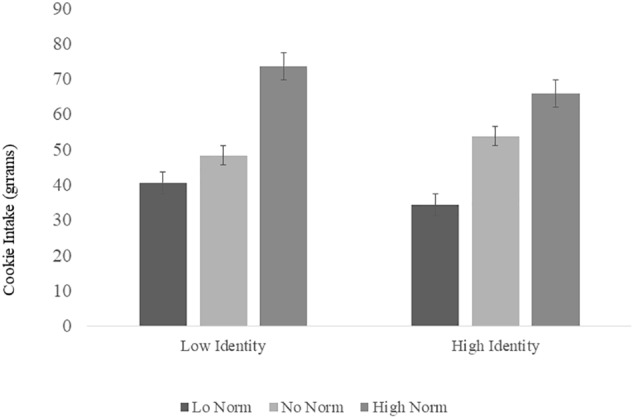
Main effect of condition, identity and interaction on cookie intake (high norm vs. no norm vs. low norm). The plotted data represent mean centered identity ± 1 SD. Low identity represents one SD below the mean, and high identity represents one SD above the mean.

There were significant modeling effects: cookie intake was higher in the high norm condition compared to the no norm condition (*b* = 18.63, *t* = 3.05, *p* = 0.003, 95%CI [6.49, 30.76]) and cookie intake was lower in the low norm condition compared to the no norm condition (*b* = -13.60, *t* = -2.27, *p* = 0.026, 95%CI [-25.53, -1.67]). There was no significant main effect of identity on cookie intake (*b* = 3.84, *t* = 0.58, *p* = 0.564, 95%CI [-9.34, 17.03]). In addition identity scores did not moderate the effect of condition on cookie intake for the no norm vs. low norm comparison (*b* = -8.161, *t* = -0.94, *p* = 0.349, 95%CI [-25.37, 9.06]) and nor did identity scores moderate the effect of condition on cookie intake for the no norm vs. high norm comparison (*b* = -9.16, *t* = -1.05, *p* = 0.299, 95%CI [-26.60, 8.27]) (Figure 1).

## Study 2

### Methods

#### Participants

We recruited 99 students from the University of Birmingham. After applying the exclusion criteria, 84 students remained in the sample (mean age of 20.5 years, *SD* = 3.2). We excluded 15 participants (7 were underweight, 4 guessed the study aims and 4 reported awareness of norms but were in the control condition). Based on calculations using GPower 3.1.0, at 85% power with a medium effect size (f^2^) of 0.15, a minimum sample of 76 participants was needed for the multiple regression analysis (with 2 tested predictors). We aimed to recruit few more participants so that the sample size was comparable to that from Study 1. As for study 1, Study 2 was advertised via an online portal and posters around campus. Students voluntarily signed up for participation and they were compensated with course credits or a $5 cash upon the completion of the study. A cover story suggested that the study was about “Vegetable Taste and Mood Status.” All criteria for taking part were as same as Study 1. The study was conducted according to the guidelines laid down in the Declaration of Helsinki and was approved by the University of Birmingham Research Ethics Committee. There was no data collection after data analysis. A *post hoc* test revealed the study was powered to detect a medium effect size (*f* = 0.15) with the final sample size.

#### Design

The study used a between-subjects design, with two conditions: message type (high intake norm vs. no intake norm) and student identity as a continuous variable. This study only included the high norm condition because increasing healthy food intake is more important for health and based on the results of Study 1 we reasoned that there would be more variability in following of the high norm condition, which is consistent with there being potential moderators of the effect. Participants were randomly allocated to one of the message conditions.

#### Remote Confederate Manipulation

In the experimental condition, participants were exposed to a sheet containing information about previous participants, including their name, age and vegetable intake. At the top of the sheet was displayed ‘Participation sheet-UoB students (University of Birmingham). This is a recording sheet of the number of vegetable sticks that each participant takes. Please fill in your information below if you are willing to take part in the study.’ A pilot study was conducted to investigate the average number of vegetable sticks that students usually consume, in order to determine the high intake norm. The total number of vegetable sticks that pilot participants consumed ranged between 6 and 60. On average, each participant consumed 21 vegetable sticks. To make a clear difference between high norm and control condition, the high norm was decided as slightly above the double of the average. Therefore, the high intake norm showed that previous participants consumed 40 to 45 vegetable sticks in total in the study.

#### Foods

Two types of vegetable were used: raw cucumber and raw red pepper. The average vegetable stick prepared for participants was about 4 cm long and weighed 5–7 g. Two separate bowls of 60 vegetable sticks (30 cucumber sticks and 30 red pepper sticks) were presented to each participant. A glass of water and napkins were also provided.

#### Measurements

In this study, we report all measures and manipulations. The same questionnaires from Study 1 were used in this study including medical history questionnaire, appetite and mood visual analog scales, habitual vegetable consumption, liking rating scales for cucumber and red pepper, Three Factor Eating Questionnaire (TFEQ), and multicomponent in-group identification questionnaire (Leach et al., 2008) (Cronbach’s alpha for the current study = 0.87). Besides that, we added a four items identification scales (0–100) in order to measure identity in a manner that is more comparable with previous studies. The four-item scale was derived from a Group Identification Scale (GIS) (Doosje et al., 1995) (Cronbach’s alpha = 0.80). We asked participants to indicate the extent to which they agree or disagree each statement: I identify with other UoB students, I see myself as a UoB student, I am glad to be a student at UoB, and I feel strong ties with UoB students. Participants’ food intake and BMI were also measured. The main outcome variable was grams of vegetable sticks consumed.

#### Procedure

We followed a similar procedure to that of Study 1. All test sessions were conducted between 10:00–12:00 and 14:00–18:00 on weekdays. Participants were asked to refrain from eating for 2 h prior to the test session. They were given a sheet containing either a high amount of vegetable consumption from previous participants or no information about others’ intake. After that, the experimenter removed the information sheet and served participants the vegetables, and participants ate for 10 min and provided liking ratings of the vegetables. Participants filled in mood questionnaires (both before and after eating), and completed the questions about habitual vegetable consumption, the student identification scales and the TFEQ. Finally, the experimenter measured the participants’ height and weight. After the session, the amount of vegetable intake was calculated and the demand check questions were completed.

### Analysis Strategy

We first examined whether there was a difference across conditions in participant age, baseline hunger and BMI, cognitive restraint, habitual vegetable intake and liking of the vegetables using independent sample *t*-tests. The two item measures on vegetable intake per day and vegetable intake yesterday were correlated. We averaged those two items to form a single measure of habitual vegetable intake. Additionally, we used correlation analysis to see if any variables correlated with vegetable intake. As in Study 1, the main planned analysis was a moderated regression analysis using PROCESS. The dependent variable was the grams of vegetables consumed.

### Results

#### Manipulation Checks

All participants correctly reported the number of vegetables eaten by previous participants as 40, 41, 42, 43, 44, or 45 sticks in the high intake norm condition (*N* = 42) and reported that no information was given in the no norm control condition (*N* = 42).

#### Participant Characteristics and Baseline Measures

There were no significant differences between conditions on the measures: age, BMI, TFEQ scores (cognitive restraint, disinhibition and hunger), liking of cucumber and red pepper, baseline appetite and baseline mood status. Details are shown in Table 2. Pearson’s correlation revealed that age was positively correlated with total vegetable intake in grams (*r* = 0.23, *p* = 0.04). Liking of cucumber was positively correlated with total vegetable intake in grams (*r* = 0.22, *p* = 0.05). Moreover, we found that liking of cucumber was positively correlated with cucumber intake in grams (*r* = 0.49, *p* < 0.001) and liking of red pepper was positively correlated with red pepper intake in grams (*r* = 0.56, *p* < 0.001). Thus, age and liking of cucumber and red pepper were controlled for the main analysis. There was no correlation between habitual vegetable intake and total vegetable intake in grams (*r* = -0.08, *p* = 0.48) and sticks (*r* = -0.11, *p* = 0.32). However, *t*-test showed that habitual vegetable intake was significantly lower in the no norm condition than in the high norm condition [*t*(81) = -1.10, *p* = 0.001], which suggested that habitual vegetable intake should be controlled as a covariate in the analysis of food intake.

**Table 2:** Participant characteristics in the high intake norm and control conditions (mean and SD).

	No norm (*N* = 42)	High norm (*N* = 42)
Age (years)	20.3 (2.6)	20.7 (3.7)
BMI	21.6 (1.9)	21.2 (1.8)
Ethnicity	White = 20 Asian = 16	White = 22 Asian = 14
Baseline hunger (0–100)	46.4 (26.3)	47.8 (27.3)
Liking of cucumber (0–100)	64.4 (25.2)	65.1 (26.1)
Liking of red pepper (0–100)	65.8 (24.1)	62.1 (30.2)
Restrained eating (0–20)	8.7 (5.1)	9.2 (5.2)
Habitual vegetable intake (servings/per day)	2.3 (1.1)	2.6 (1.7)^∗∗∗^


*^∗∗∗^p < 0.001 comparison between high norm and no norm.*

#### Total Vegetable Intake

**FIGURE 2:**
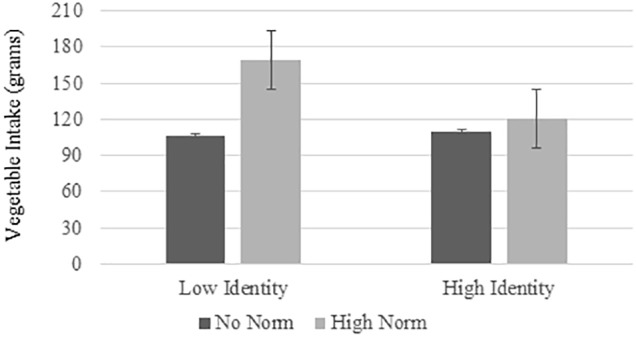
Main effect of condition, identity and interaction on effect for vegetable intake. The plotted data represent mean centered identity ± 1 SD. Low identity represents one SD below the mean, and high identity represents one SD above the mean.

There was a significant main effect of condition on vegetable intake (*b* = 36.84, *t* = 2.46, *p* = 0.016, 95%CI [7.06, 66.62]), but no significant effect of identity on vegetable intake (*b* = -16.25, *t* = -1.50, *p* = 137, 95%CI [-37.77, 5.27]) and a non-significant interaction effect between identity and condition on vegetable intake (*b* = -34.52, *t* = -1.78, *p* = 0.08, 95%CI [-73.28, 4.24]), when controlling for habitual vegetable intake as a covariate. The analysis was re-run without habitual vegetable intake as a covariate and the results were the same for the interaction effect (*b* = -37.59, *t* = -1.95, *p* = 0.06, 95%CI [-78.86, 1.41]) (Figure 2). Additionally, a separate regression analysis was conducted using identity scores from the GIS scales (Doosje et al., 1995) and this revealed the same pattern of results. There was a significant main effect of condition on vegetable intake (*b* = 35.54, *t* = 2.34, *p* = 0.022, 95%CI [5.29, 65.79]), while the identity scores from the GIS did not moderate the effect of condition on vegetable intake (*b* = -0.11, *t* = -0.08, *p* = 0.934, 95%CI [-2.71, 2.49]). Additionally, there was no significant effect of identity on vegetable intake (*b* = 0.02, *t* = 0.03, *p* = 0.973, 95%CI [-1.19, 1.24]).

## Discussion

In Study 1, we found a clear modeling effect for cookie intake in that participants who were led to believe that previous participants had eaten a large amount of cookies (a high intake norm condition) ate significantly more cookies than did participants who were given no information about how many cookies others had eaten (no norm condition). Participants who were led to believe that previous participants had eaten a small amount of cookies (a low intake norm) ate significantly less than participants in the no norm condition did. This pattern of results is consistent with previous findings that providing normative information about the intake of others affects amounts consumed (Robinson et al., 2013).

Study 2 examined the modeling of vegetable intake and it was found that young women modeled other people’s intake of vegetables (Hermans et al., 2009; Robinson et al., 2013; Vartanian et al., 2013). People who were led to believe that previous participants had eaten a lot of vegetables (a high intake norm) ate significantly more vegetables than did participants who were given no information about others’ vegetable intake (control condition). To our knowledge, Study 2 was the first study to examine modeling of vegetable intake using a remote-confederate design. Our findings are consistent with the research on live confederate modeling on nutrient-dense foods among young women (Hermans et al., 2009), in which young women adapted their intake of vegetables to that of their eating companion. The results of the Study 2 support the idea that awareness of the healthy eating habits of others may be used to promote healthy dietary choices (Robinson et al., 2013, 2014; Stok et al., 2014a).

Across two studies, we found that young women adapted their intake to be more in line with the normative intake presented. However, there was no consistent evidence to suggest that strength of identification with the norm referent group moderated this effect. This pattern of results is consistent with previous reports that modeling of eating behavior is a robust phenomenon (Roth et al., 2001; Hermans et al., 2009; Robinson et al., 2011, 2013; Cruwys et al., 2012; Vartanian et al., 2013). Overall, the results were consistent with the growing body of research suggesting that people look outward to food cues as the appropriate amount of food to consume and modeling of intake occurs even when another person is not physically present (Herman et al., 2003).

The lack of moderation of modeling by strength of identification with the group norm contrasts with previous findings that individuals model the eating behavior of others from the same social group, particularly when individuals strongly identify with the norm referent group (Cruwys et al., 2012; Stok et al., 2012). There are a number of possible reasons why identification with the norm was not a significant moderator of modeling of intake in the present studies. One reason is that our study was not sufficiently powered to detect significant interactions between condition and identity. The pattern of results for Study 1 suggests that the size of the modeling effect was very similar for both the low and high identifiers. However, in Study 2, when using the identity scores from the modified Leach et al. (2008) scale, there was a tendency for the modeling effect to be smaller in the high identifiers vs. low identifiers, although this was not the case when using the identity scores for from the GIS scale (Doosje et al., 1995). These data suggest that further investigation of the moderating effect of norm identification on modeling of intake using larger sample sizes is warranted. In addition, there may not have been not sufficient variability in the measures of in-group identification to reveal a moderating effect in our studies. Scores on the multicomponent identification scale were high on average and so it is possible that there were not sufficient participants who scored low in identification in the present sample to reveal a difference in the responses of low vs. high identifiers. Moreover, due to the sample size of the current studies we were not able to investigate the moderating role of specific subtypes of norm identification as defined by the Leach et al. (2008) scale, such as solidarity with the group or the centrality of in-group membership to the individual. Future studies might usefully examine these issues.

It is also possible that factors such as how the eating norm is conveyed and the nature of the normative information have an influence on whether or not identification with the norm referent moderates norm following. For example, moderation might be more likely if the norm is conveyed by the behavior of another present person, as in the live confederate design, rather than in the remote confederate design. Although it has been reported that the live model and remote model designs are similar (Feeney et al., 2011), there are some potential differences between the two paradigms that could help explain the present findings. Similarity or otherwise to the norm referent might be more salient in the live situation or it may be that differences between the live and remote confederate determine whether or not identification with the norm referent is a moderating factor. Modeling when there is another person present may be motivated by both the desire to behave appropriately and by the desire to affiliate with the confederate (Deutsch and Gerard, 1955; Robinson et al., 2011), whereas modeling in the remote confederate design may be more motivated by the desire to be correct (Herman et al., 2003; Robinson et al., 2013). It is possible that identification with the norm referent is more important under conditions when affiliation concerns are prominent.

Alternatively, because we did not manipulate whether the norm came from an in vs. out-group, it is possible that in the present context it was sufficient that the norm came from a relevant group and that the strength of identification with the group has no additional influence. In addition, in the present context there may have been a high degree of uncertainty about the appropriate amount to eat and the information about prior participants’ consumption provided provide a context specific norm to follow (i.e., this is how other people in this context behave). Other studies in which identification with the norm referent has been shown to be important have conveyed messages that refer to a population norm (e.g., 27% of Dutch students eat two portions of fruit per day) (Stok et al., 2012) rather than participants in a specific context (e.g., prior participants in a study), as in the present study. In addition, in the study by Stok et al. (2014b) the message first highlighted that most people do not eat enough vegetables before presenting the normative information, which may have made identification with norm more relevant in that context. Future studies could investigate the specific conditions under which identification with a norm referent moderates norm following.

A strength of our investigation is that we assessed modeling over two studies and we replicated modeling effects for both a high calorie/energy food and nutrient rich foods. However, a few limitations of the present studies should be discussed. We assessed the modeling of food intake only in young female college students. Although there is some evidence to suggest larger modeling effects for women than for men (Hermans et al., 2010), possibly because women are more concerned with how others perceive their eating behaviors (Vartanian et al., 2007), is remains unclear whether gender would interact with social identity to affect modeling of food intake. In addition, we only recruited lean participants and since previous evidence has suggested that there is an interaction between participant body weight and the model’s body weight on the degree of modeling (de Luca and Spigelman, 1979; Johnston, 2002; Hermans et al., 2008; McFerran et al., 2010), it would be of interest to examine whether group identification also interacts with weight to affect modeling. Finally, in Study 2 we only included a high norm condition and did not test the effects of a low norm condition. The reason for this was that practically speaking, increasing healthy food intake is more important for health, but it would be interesting in future studies to examine responses to a low norm condition, as was done for Study 1, to assess further the reliability of the effects.

To conclude, the results of the present studies provide evidence of robust modeling of eating behavior for both cookie and vegetable intake. Our data also suggest moderating factors such as social identity might only affect following of food intake norms under certain conditions that remain to be fully elucidated.

## Author Contributions

JL was responsible for data acquisition and analysis and drafted the initial version of the manuscript. Both authors contributed to the design of the study and interpretation of the data, were involved in critical revision of the manuscript, approved the final manuscript, and agreed to be accountable for all aspects of the work.

## Conflict of Interest Statement

The authors declare that the research was conducted in the absence of any commercial or financial relationships that could be construed as a potential conflict of interest.
